# Yellow nail syndrome following multiple orthopedic surgeries: a case report

**DOI:** 10.1186/s13256-019-2136-2

**Published:** 2019-07-01

**Authors:** Hideya Itagaki, Suzuki Katuhiko

**Affiliations:** Department of General Surgery, Honjoudaiichi Hospital, 110, Iwabuchishita, Yurihonnjou, Akita 015-8567 Japan

**Keywords:** Yellow nail syndrome, Titanium, Spinal fusion, Pleural effusion

## Abstract

**Background:**

Yellow nail syndrome is a rare condition associated with a triad of symptoms: yellow nails, lung lesions, and lymphedema. We report a case of yellow nail syndrome caused by titanium exposure from multiple artificial joint replacements.

**Case presentation:**

A 78-year-old Asian woman presented to our outpatient department with chief complaints of cough, fever, and nausea. The patient was hospitalized for observation because of the presence of hypoxemia and bilateral pleural effusion. Her medical history included knee joint replacement and two spinal fusion surgeries. Her physical examination conducted following hospitalization revealed yellow nails on both hands and feet. This finding, combined with the observation of bilateral pleural effusion, raised suspicion for yellow nail syndrome. Blood analysis yielded negative results, as did the tests for sputum culture, interferon liberation, pleural effusion culture, and pleural effusion cytology. Pleural histopathological analysis and imaging yielded negative results. Considering the possibility of titanium exposure from artificial joints based on the patient’s medical history, we examined a chest radiograph obtained before the second spinal fusion surgery; however, no pleural effusion was observed. Pleural effusion was observed, however, following the surgery. On the basis of these findings, the patient was diagnosed with yellow nail syndrome due to titanium exposure.

**Conclusions:**

Clinicians should examine the nails of patients with unexplained pleural effusion. Moreover, they should inquire about titanium exposure when obtaining the patient’s medical history.

## Background

Yellow nail syndrome (YNS) is a rare condition that was first identified by Samman *et al.* in 1964. YNS is characterized by a triad of symptoms: yellow nails, lung lesions, and lymphedema [1]. Although the condition’s etiology remains unclear, YNS is associated with cancer and autoimmune diseases. YNS may also develop following dental treatment or titanium exposure from cardiac pacemakers or artificial joints [2]. Previously published case reports of YNS due to titanium exposure could not confirm the number of surgeries undergone by patients. Our patient had undergone joint replacement twice without the development of YNS; therefore, attributing titanium exposure as the cause of YNS following the third surgery was difficult because no disease onset was observed following the initial two surgeries. We report an interesting case of YNS caused by titanium exposure from multiple artificial joint replacement surgeries.

## Case presentation

A 78-year-old Asian woman presented to our outpatient department with chief complaints of coughing and fever. Her cough had persisted for several weeks, and her fever had developed on the previous day. The patient’s medical history included asthma and sinusitis. Although her sinusitis had been treated several years prior, she had not received treatment before hospitalization. The patient’s surgical history included knee joint replacement and two spinal fusion surgeries; the second spinal fusion had been performed 3 months before the current consultation.

**Fig. 1:**
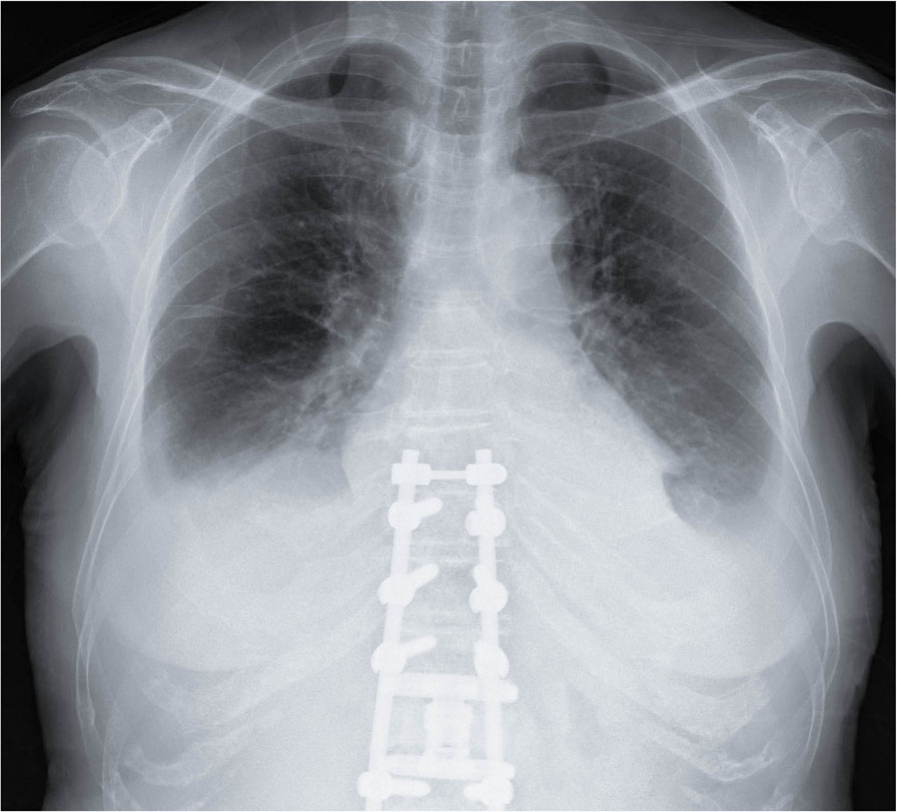
Bilateral pleural effusion was detected on a chest radiograph at the time of hospitalization

**Fig. 2:**
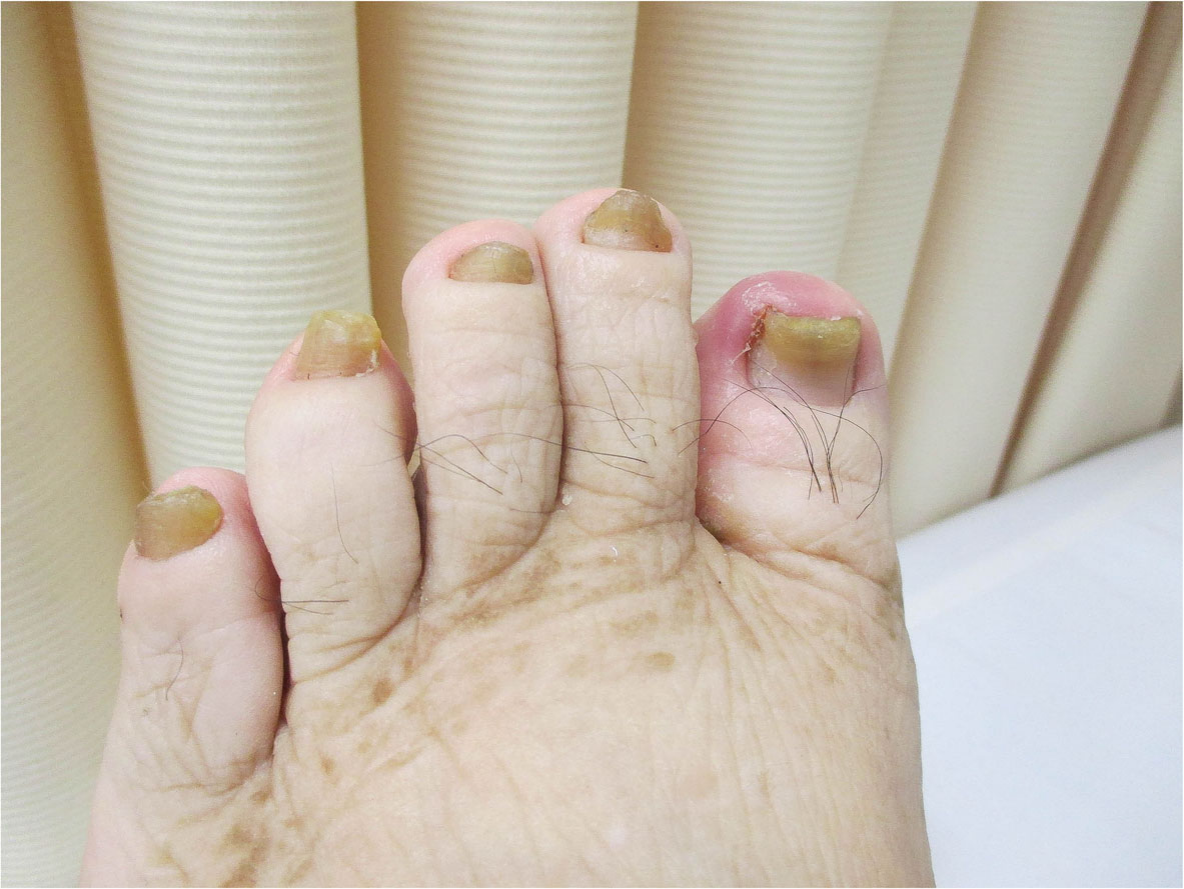
Yellow toenails

**Fig. 3:**
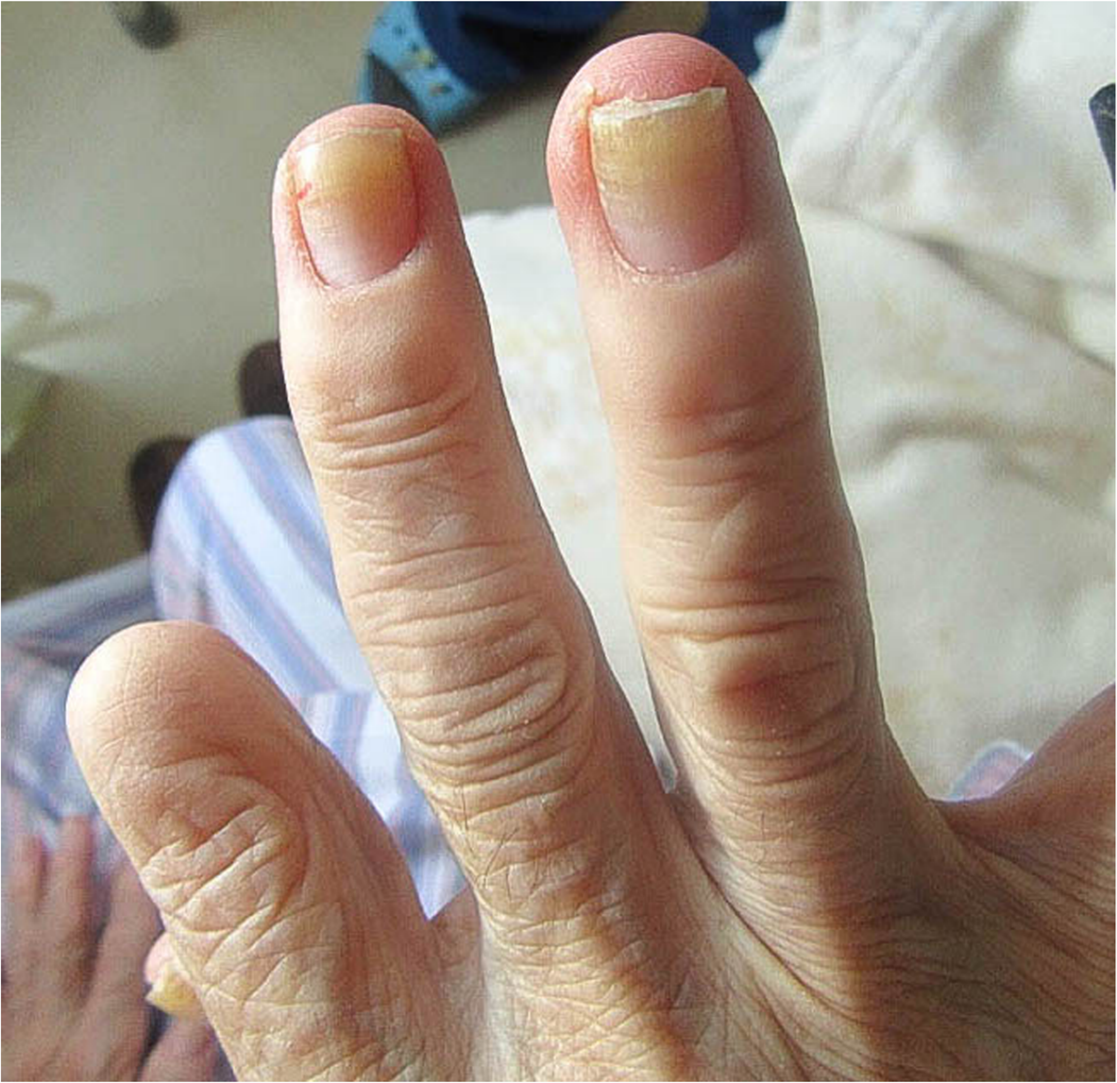
Yellow fingernails. The right index finger distal interphalangeal joint (DIP) and beyond are lost due to trauma

**Fig. 4:**
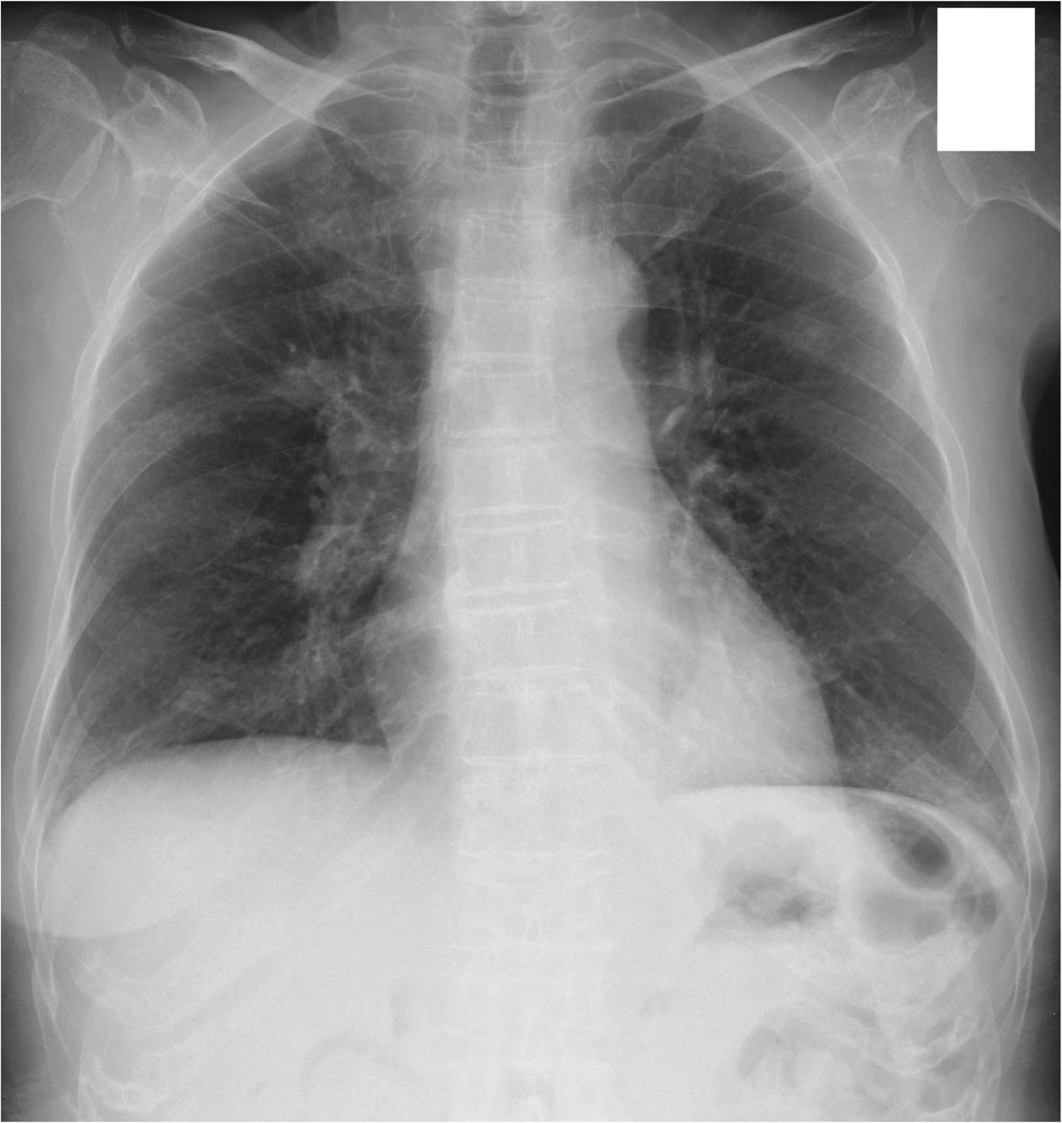
Chest radiograph obtained prior to the second spinal fusion showing no pleural effusion

Physical examination revealed hypoxemia, and auscultation revealed bilateral chest crackles with no sign of heart failure. Bilateral pleural effusion was detected on a chest radiograph (Fig. 1). The patient developed yellowing of her fingernails and toenails following hospitalization (Figs. 2 and 3). This finding, combined with the patient’s pleural effusion and sinusitis, led to suspicion for YNS. Bilateral dorsum pedis lymphedema was confirmed during hospitalization. Blood analysis revealed a slight increase in inflammation. However, the patient’s test results for rheumatoid factor and anti-cyclic citrullinated peptide antibody were negative. Her thyroid parameters and levels of soluble interleukin-2 receptor were normal. Her sputum culture and interferon-γ release assay results were negative. The result of her bacterial culture of pleural effusion was negative. Computed tomography failed to confirm the presence of a malignant tumor. A chest radiograph (Fig. 4) obtained prior to the second spinal fusion procedure showed no pleural effusion. However, pleural effusion appeared 1 month after the second surgery. On the basis of these findings, the patient was diagnosed with YNS due to titanium exposure.

**Fig. 5:**
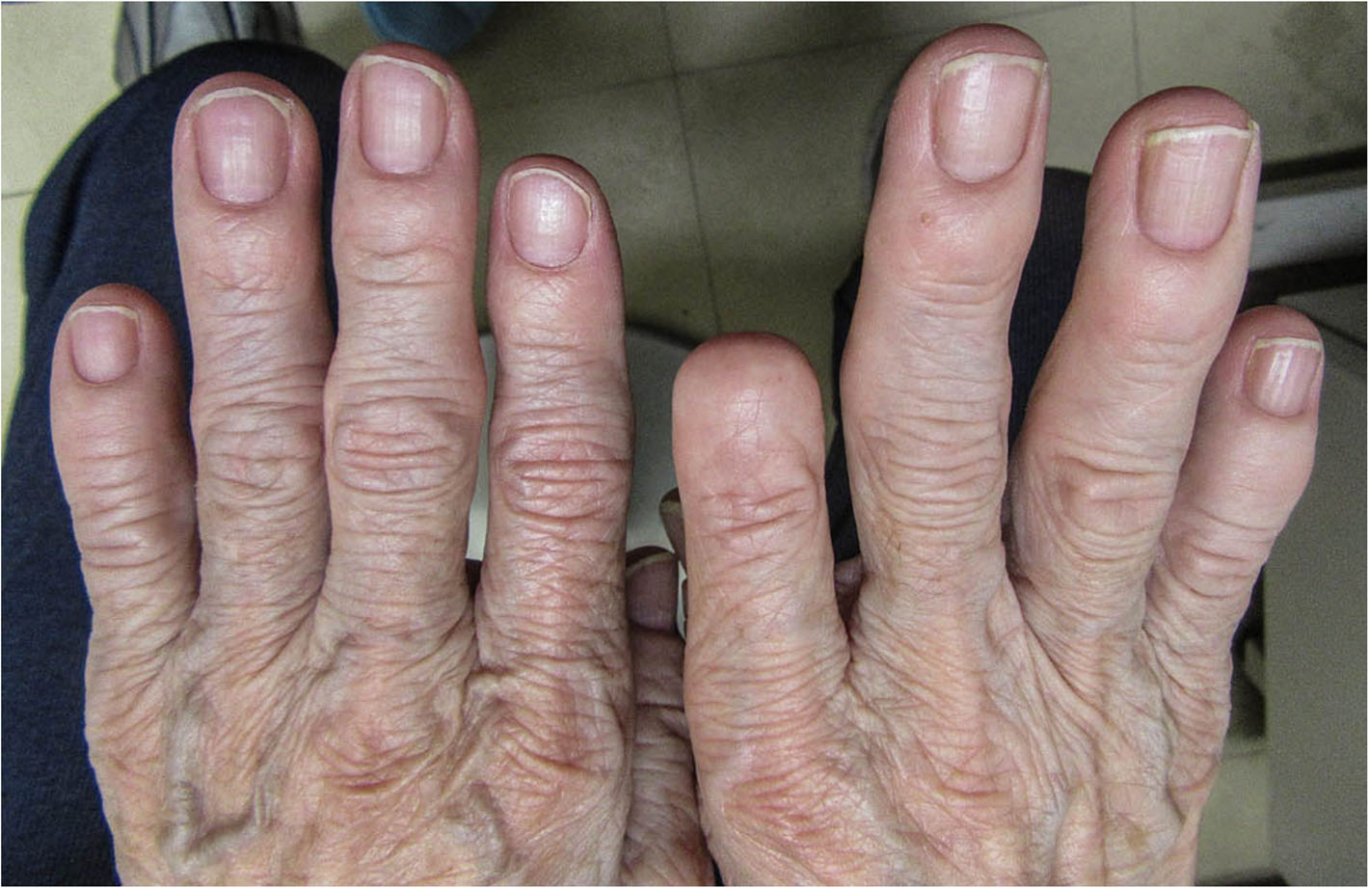
Improvement in the thickness of the nails on the hands

After diagnosis, vitamin E was administered for more than 1 year. After a half-year of vitamin E administration, improvement in the thickness of the nails on the patient’s hands was observed (Fig. 5), but no effect was seen for the pleural effusion. Pleural effusion also failed to respond to pleurodesis. Pleural effusion drainage was therefore performed regularly. Currently, the patient visits our clinic every 1–2 months and undergoes chest radiography. Pleural drainage is performed if there is an increase in pleural fluid.

## Discussion

YNS is a rare condition with an estimated incidence of 1 in 1,000,000 [1, 3]. It typically affects individuals aged between 50 and 60 years, with both genders being similarly affected [4]. The etiology of YNS remains unclear, but it may involve an acquired dysfunction of the lymphatic circulation.

Nail abnormalities are found in 90–100% of patients with YNS [3], and they include yellow discoloration, nail-plate thickening, detachment, delayed growth, transverse ridges, excessive curvature, and pains. YNS generally affects all the nails of both hands and feet. Respiratory lesions are observed in 50–70% of patients with YNS, with associated chronic cough, pleural effusion, bronchiectasis, recurrent pneumonia, and chronic sinusitis. The most common YNS symptom is chronic cough, found in 56% of patients with YNS [4]. Pleural effusion is bilateral in 68.3% patients and exudative in 94.7%, with lymphocytes predominant in the exudate (96%) [5]. Lymphedema is observed in 30–80% of patients with YNS [3]; it appears in both lower extremities, usually below the knee. Increased lymphedema is considered to be indicative of the excessive accumulation of lymphocytes and fiber formation that is caused by the stimulation of fibroblasts and adipocytes.

The presence of two symptoms from among the triad of yellow nails, lung lesions, and lymphedema is sufficient for a diagnosis of YNS because the three characteristic symptoms do not typically appear simultaneously. All three YNS symptoms are present in 27–76% of cases [6, 7]. Our patient’s case of YNS was diagnosed on the basis of the presence of yellow nails and respiratory lesions (chronic cough, bilateral pleural effusion, and sinusitis) as well as lymphedema noted on both feet during hospitalization. The patient thus presented with all three symptoms over time. Pleural effusion was bilateral and exudative with lymphocyte-predominant white blood cells; this finding was consistent with YNS.

YNS has been associated with various diseases, including cancer, autoimmune dysfunction, immunodeficiency, nephrotic syndrome, hyperthyroidism, hypothyroidism, rheumatoid arthritis, and tuberculosis [3]. Moreover, titanium exposure has also been implicated [8]. Berglund *et al.* [8] used energy-dispersive X-ray fluorescence to analyze nail samples from 30 patients with at least one YNS symptom, and the results showed that high levels of titanium (1.1–170 μg/g nail) were present. The authors also reported that gold or amalgam (alloys of mercury and other metals used as dental filling material) could induce galvanic corrosion of titanium, resulting in the release of titanium ions. The removal of gold teeth from four patients wearing titanium implants resulted in the complete resolution of symptoms. Titanium removal is therefore recommended. However, for cases in which titanium removal is difficult, further research is necessary to determine an alternative approach to treatment.

Previous studies have demonstrated that vitamin E supplementation [9], antifungal agents [10], and clarithromycin [11, 12] are effective in the treatment of YNS. In our patient’s case, oral administration of vitamin E was recommended as treatment because the removal of titanium was difficult. Vitamin E may be administered locally (topical vitamin E) or orally (oral vitamin E). Local administration prevents the deposition of lipofuscin, which causes nail yellowing, and is therefore considered to be an effective treatment for nail symptoms [13]. Oral administration also reportedly prevents the deposition of lipofuscin production that is possibly responsible for nail yellowing [14]. Symptoms have reportedly been resolved with oral administration of 800 mg of vitamin E as a first dose, followed by 400 mg every 24 h [9]. Other researchers have reported an improvement in symptoms with the simultaneous oral administration of vitamin E and antifungal drugs [10, 15]. However, using antifungal drugs alone remains controversial. Tosti *et al.* administered itraconazole to patients with YNS but did not report on its efficacy [16]. In our patient’s case, although oral vitamin E improved the patient’s nail color, it had no effect on pleural effusion. Therefore, we proceeded with pleurodesis. Surgical intervention is an effective treatment for pleural effusion. According to Valdés *et al.*, the effective rates of pleurodesis, decortication/pleurectomy, and pleural–peritoneal shunt for pleural effusion in patients with YNS were 81.8%, 88.9%, and 66.7%, respectively [5]. For pleurodesis, talc [17] and OK-432 [18] are generally used as pleurodesis adhesion materials. In our patient’s case, OK-432 was used for pleurodesis. However, pleurodesis failed to improve the pleural effusion; therefore, we regularly performed pleural effusion drainage.

## Conclusions

YNS, which may be caused by titanium exposure, is a rare complication of multiple joint replacement surgeries. A patient’s nails should be examined in the case of an unexplained pleural effusion, and the surgical history, particularly the number of operations the patient has undergone, should be evaluated. As the population ages and the number of patients with artificial joints increases, the incidence of YNS may also increase. Surgical intervention is effective in the treatment of pleural effusion in patients with YNS.

## Data Availability

Data sharing is not applicable to this article, because no datasets were generated or analyzed during the current study.

## Abbreviation

YNS: Yellow nail syndrome
